# Evaluation of the Molding Helmet Therapy for Japanese Infants with Deformational Plagiocephaly

**DOI:** 10.31662/jmaj.2020-0006

**Published:** 2021-01-15

**Authors:** Ako Takamatsu, Makoto Hikosaka, Tsuyoshi Kaneko, Masashi Mikami, Akiko Kaneko

**Affiliations:** 1 Division of Plastic Surgery, National Center for Child Health and Development, Tokyo, Japan; 2 Biostatistics, Clinical Research Center, National Center for Child Health and Development, Tokyo, Japan; 3 Bona Dea Clinic, Kanagawa, Japan

**Keywords:** deformational plagiocephaly, deformational brachycephaly, craniosynostosis, molding helmet therapy, plagiocephaly, positional skull deformity, brachycephaly, infant

## Abstract

**Introduction:**

Deformational plagiocephaly (DP) is cranial flattening on one side of the back of the skull produced by an extrinsic force on the intrinsically normal skull. When the flattening is symmetrical, the deformity is called deformational brachycephaly (DB). In the US, its prevalence has increased since the “Back to Sleep” campaign by the American Association of Pediatrics. Helmet therapy is reported to be effective in improving head deformity by multiple studies, but there are few evidences from Japan. The purpose of this study is to investigate the safety and efficacy of helmet therapy for DP, and the feasibility of introducing this treatment to the clinical setting in Japan.

**Methods:**

This was a single-arm, retrospective, nonrandomized study. Data were collected on infants who visited the “Clinic for Baby’s Head Shape” in the National Center for Child Health and Development, Tokyo, Japan, between 2011 and 2014. Improvements in Argenta classification, cranial asymmetry (CA), and cranial vault asymmetry index (CVAI) were evaluated. The relationships between CA and influencing factors were evaluated using a linear mixed-effects model.

**Results:**

Three hundred eighty-seven infants (273 boys and 114 girls; average age, 4.7 months) visited the clinic during the period, and 159 patients who completed the helmet therapy were analyzed. There were statistically significant improvements in Argenta classification, CA, and CVAI. Almost all of the parents reported increased sweating and mild skin irritation, but no adverse events necessitated the cessation of helmet therapy, except for one patient with increased sweating.

**Conclusions:**

Helmet therapy is safe and effective in treating DP and is feasible to introduce to the clinical setting in Japan. Through the distribution of knowledge regarding the etiology and treatment of head deformity, earlier detection and an evidence-based approach to head deformity are expected in the future.

## Introduction

Deformational plagiocephaly (DP) is defined as cranial flattening on one side of the back of the skull produced by extrinsic forces on the intrinsically normal skull ^(1)^. This condition occurs primarily in infants who consistently favor turning their head to one side during the first few months of life. Various risk factors such as male gender, multiple pregnancy, premature delivery, assisted delivery, and torticollis ^(2)^ have been reported. Once the flattening has progressed to some extent, the spontaneous correction of the deformation is difficult even after the preferred head position has disappeared because the extrinsic force is continuously applied to the flattened area on a resting surface ^(3), (4)^. Clinical findings consist of flattening of one side of the back of the skull, anterior position of the ear on the same side, asymmetry of the forehead or cheek, and in severe cases “rhomboid-shaped head” as observed from the top. Symmetrical occipital flattening denotes deformational brachycephaly (DB).

### History of the treatment of DP

In 1992, the American Academy of Pediatrics recommended not to place infants in the prone position during sleep to reduce the incidence of sudden infant death syndrome ^(5)^. Before the recommendation, many infants were laid in the prone position during sleep, and the incidence of DP was estimated as 0.3%. After the recommendation, the prevalence of DP has increased to as high as 48% ^(6), (7)^. Infants with DP/DB are initially provided with repositioning or physical therapy, and for severe deformity, helmet therapy is applied ^(8)^. The Food and Drug Administration in the US has approved more than 50 types of helmets for the treatment of DP ^(9)^.

The efficacy of helmet therapy on DP/DB is reported in various studies, mostly case series and case-control studies ^(10), (11)^. In 2016, an evidence-based guideline was issued by the Joint Committee of the American Association of Neurological Surgeons and the Congress of Neurological Surgeons and American Academy of Pediatrics ^(12)^. It states that helmet therapy improves severe deformity in a shorter time compared to repositioning and physical therapy. In general, the efficacy of helmet therapy for DP/DB is accepted in the US and European countries.

### History in Japan

In Japan, infants have traditionally been laid on their backs, and DP/DB has been commonly observed and culturally accepted. The incidence of DP/DB in Japanese infants is unknown but is expected to be much higher than that in the US and European countries. Most parents were not provided with evidence-based care for the head shape of their infants. Many pediatricians and gynecologists provide conservative explanations that children’s head shape would improve naturally. There are many nursing care products, such as doughnut-shaped pillows, that claim effectiveness in improving infants’ head shape without sufficient evidence.

More recently, parents are becoming more and more conscious regarding their infants’ head shape. The number of parents who visit pediatric neurosurgeons or plastic surgeons to consult regarding their babies’ head shape is dramatically increasing. However, there are few reports regarding the treatment of DP/DB in Japan ^(13)^, and evidence is lacking concerning the safety and efficacy of helmet therapy in Japanese infants with DP/DB.

The objectives of this study are to examine the safety and effectiveness of helmet therapy on DP and to evaluate the feasibility of introducing the treatment to the clinical setting in Japan.

## Materials and Methods

This retrospective cohort study was approved by the institutional review board (no. 511) at the National Center for Child Health and Development (Tokyo, Japan). The included patients were those who visited the Clinic for Baby’s Head Shape between October 28, 2011, and March 26, 2014, diagnosed as DP and completed helmet therapy.

### Evaluation and treatment algorithm

The clinic is run by three board-certified plastic surgeons and one orthotist. It is open to patients with head deformity with a referral letter from primary care pediatricians.

**Figure 1.**
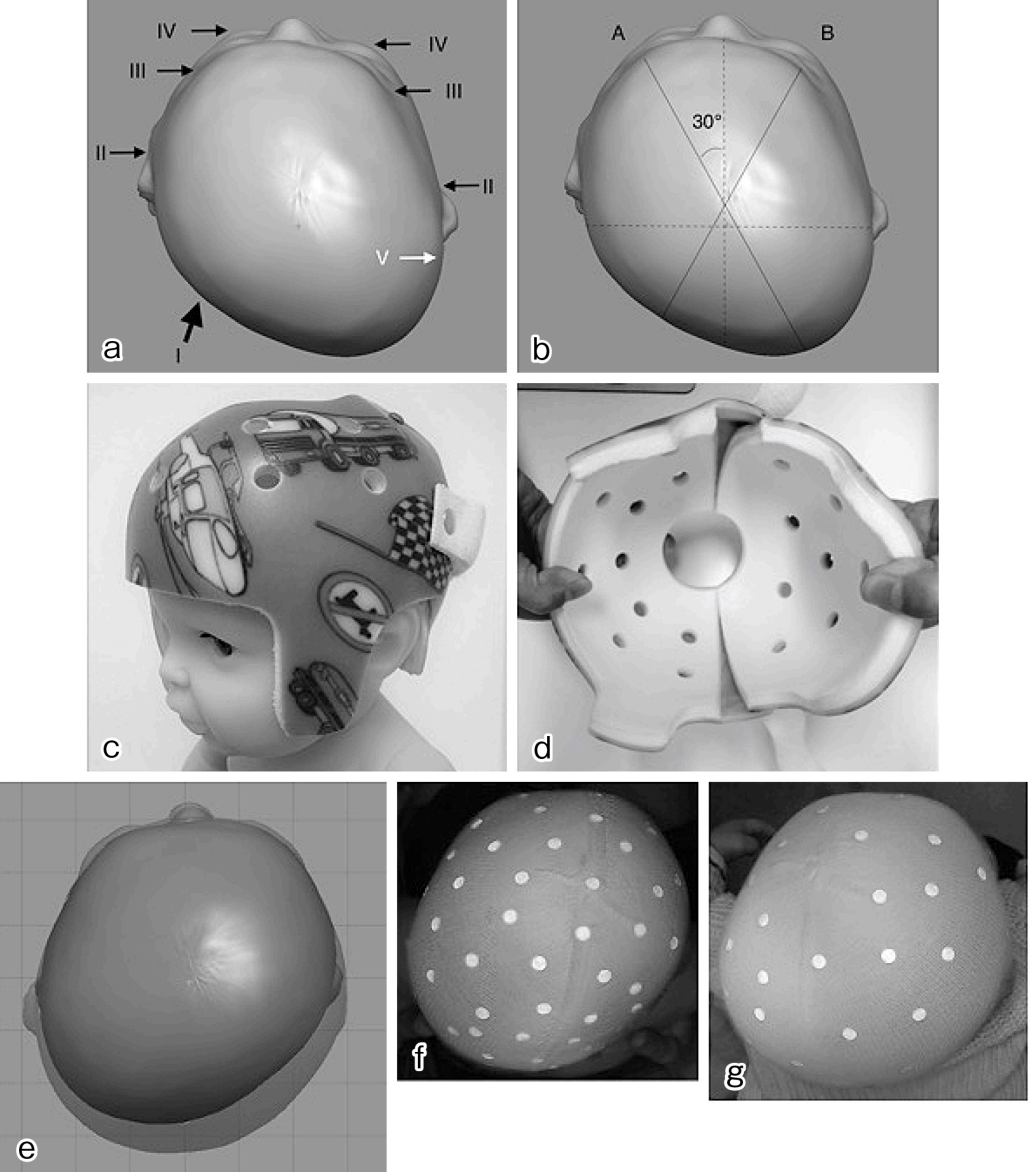
a) Argenta’s classification. TYPE 1: posterior asymmetry; TYPE 2: anterior ear shift on the side of the occipital flattening; TYPE 3: frontal asymmetry; TYPE 4: facial asymmetry; TYPE 5: temporal bossing or posterior vertical cranial growth. b) Loveday’s anthropometric cranial vault measurement. A: diagonal on the left anterior to right posterior drawn 30° from the midline of the head; B: right anterior to left posterior; cranial asymmetry (CA) = |A − B|; cranial vault asymmetry index (CVAI) = |A − B|/A or B (whichever is shorter) × 100; cephalic index (CI) = cranial width/cranial length × 100 c) Michigan Cranial Reshaping Orthosis d) The helmet shell is elastic plastic, and inside is covered with polyethylene foam with a low risk of causing allergic reactions. It consists of two pieces and is easy to adjust for infants’ growing head. It weighs less than 200 g and is well tolerated by babies. e) Comparison image of scanned data before and after treatment in a typical case (no. 127, 4 months old, male). f) Photograph of the same patient taken from the top of the head before treatment. CA = 17 mm, CI = 98.5. g) After treatment. CA = 7 mm, CI = 89.7

Head deformity was evaluated according to the modified Argenta classification ^(14)^ and anthropometric measurements (Figure 1a and b). The anthropometric measurements of the cranial vault were obtained using a craniometer and tape measure. The reliability of these evaluation methods is reported in past studies ^(15), (16), (17)^. According to the method proposed by Loveday et al. ^(18)^, head circumference, cranial asymmetry (CA), and cephalic index (CI) were used. CA and cranial vault asymmetry index (CVAI) describe the severity of asymmetry. We defined CA of less than 5 mm as normal skull, 5-9 mm as mild, 10-14 mm as moderate, 15-19 mm as severe, and ≥20 mm as very severe deformity. To enable a comparison with past reports, we also described CVAI, and CVAI of less than 3.5% was defined as normal, 5%-6% as mild, 7%-9% as moderate, 10%-13% as severe, and ≥14% as very severe ^(14)^. CI describes the brachycephalic or dolichocephalic tendency. CI of less than 79 was defined as dolichocephaly and greater than 94 as brachycephaly based on the Japanese standard ^(19)^.

**Figure 2.**
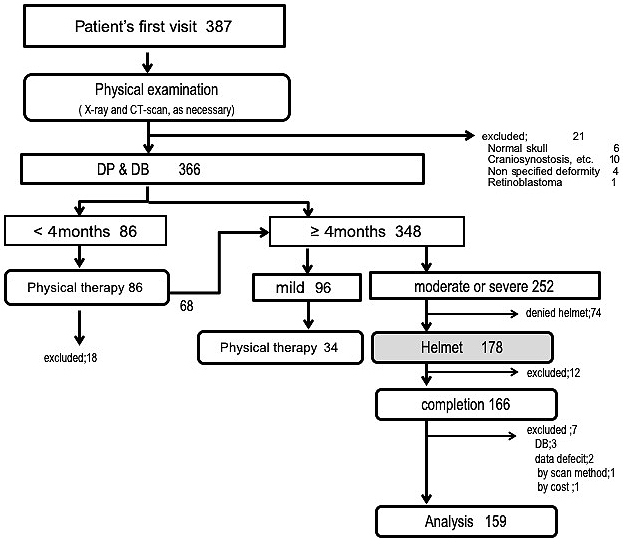
Treatment algorithm.

The patients were evaluated and treated according to the algorithm (Figure 2). The criteria for helmet therapy were Argenta ≥ II. When the parents consented with the treatment, helmet therapy was initiated.

For helmet production, infant’s head was scanned using a surface scanner (OMEGA Scanner, Ohio Willow Wood Company, Ohio, USA). The helmet was designed by a single plastic surgeon (T.A.) using specialized software (OMEGA Tracer, Ohio Willow Wood Company, Ohio, USA) supervised by a senior plastic surgeon (T.K.). The helmet (Michigan Cranial Reshaping Orthosis, Danmer Products, Michigan, USA) was fabricated in the US and sent to us (Figure 1c and d) ^(20)^. The family was instructed to wear the helmet for 23 h a day after setting a break-in period of 7-14 days, during which the wearing time is gradually increased, and to visit the clinic after 3 to 4 weeks for adjustment. The helmet was continually used until the helmet was tight or until the parents were satisfied with the infant’s head shape. At the completion of the treatment, the patient was re-evaluated using the Argenta classification, anthropometric measurements, and surface scanner. For the time period of this study, the cost of the helmet was partially covered by our research fund, and patients paid 100,000 Japanese yens.

### Statistical analysis

The primary outcomes were improvement in Argenta classification or CA. Because the treatment was considered successful if either of the two criteria improved, we set the statistical significance level in each test at 0.025, which is an adjustment by Bonferroni correction. Improvements in CVAI and CI were also evaluated.

To consider the influencing factors of the change in CA, we used a linear mixed-effects model. The outcome was pre-post CA, where pre and post mean the start and end of helmet therapy. The fixed effects were the treatment period (pre is day 0), pre-Argenta classification, pre-age, pre-head circumference, and the interaction term of the treatment period and pre-Argenta classification. The random effect was a subject-specific intercept. Because Argenta classification VI (brachycephaly) was considered to be not continuous with I to V (plagiocephaly) in severity, this was excluded from the analysis.

SAS Version 9.4 (SAS Institute Inc., Cary, North Carolina, USA) was used for Bowker test and a linear mixed-effects model. EZR (Saitama Medical Center, Jichi Medical University, Saitama, Japan) ^(21)^, which is a graphical user interface for R (The R Foundation for Statistical Computing, Vienna, Austria), was used for the other tests. As a reference, statistical significance level was set at 0.05, except for the primary outcome analysis.

## Results

During the period of 2 years and 4 months, 387 infants (273 boys and 114 girls) visited the clinic. The average age at first presentation was 4.7 months (1 to 13 months; SD, 1.79).

### Differential diagnosis and associated comorbidities

Twenty-one patients were ruled out of DP/DB. Six patients were diagnosed as normal skull without DP (CA < 5 mm). Ten patients were strongly suspected of craniosynostosis after X-ray and/or CT (four patients were definitively diagnosed as craniosynostosis, one patient was sent back for further evaluation to the referring hospital, another patient was lost to follow-up before further evaluation, and four patients were ruled out of craniosynostosis but declined helmet therapy). Four patients were diagnosed as head deformity other than DP/DB (macrocephaly, brachycephaly due to Down syndrome, calcified cephalohematoma, and dolichocephaly due to a transverse uterine position). Another patient with retinoblastoma was excluded because the condition may have influenced the head shape (Figure 2).

Among 366 patients diagnosed as DP/DB, there were 48 patients (51 cases) with associated comorbidities, with muscular torticollis as the most frequent (19 patients) (Table 1).

**Table 1.** Demographics and Associated Comorbidities. The List of Comorbidities Include Duplicate Patients with Multiple Comorbidities.

	No.of Patients			(%)
Total patients who visited the clinic	387		100%	
Normal skull		6		(1.6%)
Pathological Conditions		13		(3.4%)
Craniosynostosis (incl.susp)		10		(2.6%)
Sagittal synostosis		1		(0.3%)
Right coronal synostosis		1		(0.3%)
Metopic synostosis		1		(0.3%)
Bilateral almboid synostosis		1		(0.3%)
Lost to follow up		2		(0.5%)
Ruled out		4		(1.0%)
Other head deformities		4		(1.0%)
Macrocephaly		1		(0.3%)
21 trisomy		1		(0.3%)
Dolichocephaly due to transverse uterine position		1		(0.3%)
Calcified Cephalohematoma		1		(0.3%)
DP & DB		366		100%
Assoicated with comorbidities		48		(13.1%)
Torticollis		19		(5.2%)
Ear deformities (microtia, folded ear, crypt ear)		11		(3.0%)
Cleft lip /palate		4		(1.1%)
Calcified Cephalohematoma		4		(1.1%)
Dolichocephaly due to breech position		5		(1.4%)
Strabismus		1		(0.3%)
Congenital nystagmus		1		(0.3%)
Cerebral infarction		1		(0.3%)
Atlantoaxial subluxation		1		(0.3%)
Hemangioma		1		(0.3%)
CHARGE Syndrome		1		(0.3%)
Connective Tissue Disease with heart failure (unspecified)		1		(0.3%)
Acetabular dysplasia		1		(0.3%)

### Patients

Among the 366 patients with DP or DB, 86 patients were younger than 4 months old, and physical therapy was introduced. Among them, 68 failed to improve until 4 months old and proceeded to re-evaluation for helmet therapy. Among 348 patients with DP or DB who were older than 4 months old (including the 68 after physical therapy), 252 were diagnosed as more severe than mild, and helmet therapy was introduced to 178 patients whose parents were willing for the treatment. Twelve patients discontinued the therapy before completion. The reasons for discontinuation were unknown in nine patients because of lost to follow-up and one in each of the following: profound sweating and crying at night, mother going back to work, and death due to heart disease. Three patients with DB without plagiocephalic deformity, two patients whose data on treatment period were missing, one patient whose helmet was manufactured from CT data, and another patient whose cost was not covered by our research fund were excluded. The following analysis is focused on 159 patients who completed helmet therapy with full set of data (Figure 2).

The average age at the start of helmet therapy was 24.1 weeks (SD, 5.0), and the average treatment period was 21.2 weeks (SD, 5.3).

### Primary outcomes

Before the start of helmet therapy, type IV was most frequent in Argenta classification (69 patients, 43%), and the average CA was 16.3 mm. After the treatment, a statistically significant improvement was observed in both Argenta classification (p < 0.001, Bowker test) and CA (p < 0.001, paired t-test). Type I was most frequently observed (51 patients, 32%), and the average CA was 7.7 mm. A statistically significant improvement was also observed in CI and CVAI (P < 0.001, paired t-test) (Table 2, Figure 1e, f and g).

**Table 2.** Treatment Results. Values are Number of Patients Unless Stated Otherwise. Values before and after are Compared Using Paired T-test.

Outcomes	Classification	Definition	Before Helmet	(%)	After Helmet	(%)	p-value
Argenta type	non-DP		0	0.00%	24	(15.1%)	
	I		7	(4.4%)	51	(32.1%)	
	II		8	(5.0%)	50	(31.4%)	
	III		49	(30.8%)	15	(9.4%)	
	IV		69	(43.4%)	18	(11.3%)	
	V		15	(9.4%)	1	(0.6%)	
	VI		11	(6.9%)	0	(0.0%)	
	average: type (SD)		3.7	(SD 1.1)	1.7	(SD 1.2)	p < 0.001
CA	normal	0-4	0	(0.0%)	34	(21.4%)	
	mild	5-9	7	(4.4%)	79	(49.7%)	
	moderate	10-14	46	(28.9%)	74	(46.5%)	
	severe	15-19	66	(41.5%)	6	(3.8%)	
	very severe	= > 20	40	(25.2%)	0	(0.0%)	
	average: CA (SD)		16.3	(SD 4.2)	7.7	(SD 3.5)	p <0.001	
CVAI	normal	0-4	2.0	(1.3%)	68.0	(42.8%)	
	mild	5-6	3	(1.9%)	27	(17.0%)	
	moderate	7-9	34	(21.4%)	59	(37.1%)
	severe	10-13	58	(36.5%)	5	(3.1%)	
	very severe	= > 14	62	(39.0%)	0	(0.0%)	
	average: CVAI (SD)		12.9%	(SD 3.6)	5.4%	(SD 2.4)	p < 0.001	
CI	dolicho	= < 78	8	(5.1%)	7	4%	
	meso	79-94	70	(44.3%)	127	80%	
	brachy	= > 95	4	(46.8%)	23	9%	
	average: CI (SD)		93.5	(SD 8.3)	88.7	(SD 5.6)	p < 0.001	

### Influencing factors

Table 3 shows the coefficients of the fixed effects and the p-value derived by Wald tests in a linear mixed-effects model. The treatment period was the only factor considered as statistically significant (p < 0.001). The fixed effect shows that the CA is likely to decrease by 0.40 mm (which means the improvement in deformity) in point estimation if the infants receive helmet therapy for one week. Similarly, the CA is likely to increase (which means less improvement in deformity) if the treatment starts later (the fixed effect of age increases) or the head circumference is large at the start of helmet therapy, although these two factors did not reach statistical significance.

**Table 3.** Multivariate Linear Regression Analysis. Treatment Time was the Only Factor That Reached Statistical Significance.

		coefficient	standard error	p-value
intercept		3.55	6.98	0.6125
treatment period (week)		-0.40	0.08	<.0001^*^
age (week)		0.09	0.06	0.1086
head circumference (mm)		0.02	0.02	0.2389
pre-Argenta	1	0		
	2	-0.55	2.03	0.7871
	3	2.06	1.60	0.1977
	4	2.32	1.57	0.1418
	5	1.71	1.80	0.3427	
treatment period (week) × pre-Argenta	1	0		
	2	-0.02	0.12	0.8612
	3	0.00	0.09	0.989
	4	0.05	0.08	0.5216
	5	0.10	0.09	0.2753

**Figure 3.**
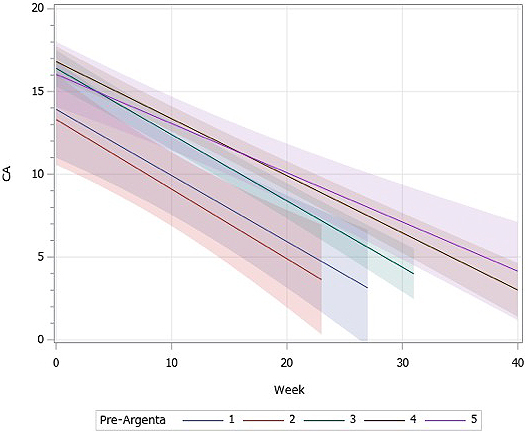
Multivariate linear regression analysis of influencing factors on CA: Each band describes the 95% confidence intervals of CA along with treatment time, compared among different pre-Argenta groups. The patients with larger pre-Argenta tended to have greater CA at the start of the therapy, improve slowly, and end in larger CA at the end of the therapy, as shown with a higher intersection and gentler slope.

Figure 3 shows the change in CA along the treatment period, stratified among pre-Argenta groups. The line shows the least-square means of each pre-Argenta group, and the band shows its 95% confidence intervals. The lines with larger pre-Argenta tended to have higher CA at the start of treatment and gentler slopes. This result could also be confirmed by the coefficients of pre-Argenta and the interaction term. It shows that patients with larger pre-Argenta necessitated a more extended treatment period to reach the same degree of CA compared to the less deformed patients. The range of the treatment period where the bands do not overlap between type II and types IV & V shows that there is a statistically significant difference between the groups.

**Figure 4.**
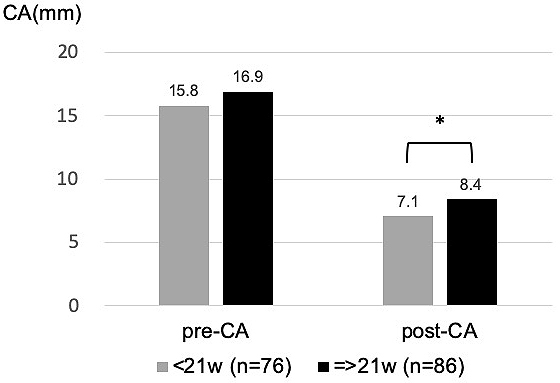
Influence of treatment period on CA: CA after helmet therapy was statistically larger in the group whose treatment period was equal to or longer than 21 weeks, which was the average duration of helmet therapy. (*p = 0.02, t test)

**Figure 5.**
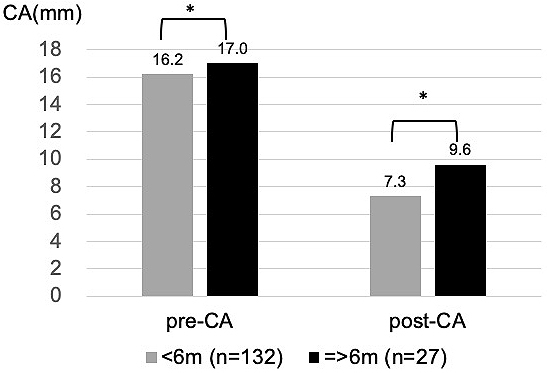
Influence of age at the start of helmet therapy on CA: CA after helmet therapy was statistically greater if the therapy was started at equal to or older than 6 months old. (*p < 0.001, t-test)

**Figure 6.**
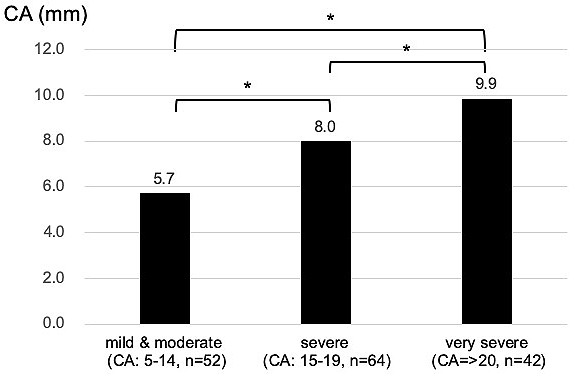
Influence of the CA before helmet therapy on CA after therapy: CA after helmet therapy tended to be larger in the group with larger CA before therapy. This difference and trend were statistically significant (*p < 0.001, one-way ANOVA. p < 0.05 for all post hoc analysis after Bonferroni adjustments. p < 0.001, Jonckheere-Terpstra test for testing the trend).

The relationships between individual influencing factors and CA were also investigated. CA after helmet therapy was statistically larger (severe deformity) in the group whose treatment period was equal to or longer than 21 weeks, who started helmet therapy at older than 6 months, or whose CA before treatment was larger (Figure 4, 5 and 6).

There was no clear relationship between the head circumference at the start of the therapy and final CA. This was true after adjusted for age and sex.

### Safety of orthotic therapy

Almost all of the parents reported increased sweating, mild skin irritation, or rash associated with helmet therapy, but these adverse events were relieved over time, with some requiring ointment treatment. One patient discontinued the use of the helmet because of profound sweating and was excluded from outcome analysis. In one patient, skin blister was observed because of insufficient break-in period, but helmet therapy was resumed after 5 days of rest. No skin ulcer was observed. A total of nine unplanned additional visits were required in six patients for adjustment of the helmet. No additional helmet was required in all of the patients.

## Discussion

### Primary outcomes

There was a statistically significant improvement both in Argenta classification and CA (CVAI), and helmet therapy was effective in treating DP.

Helmet therapy is reported to improve severity in Argenta classification in past studies. According to Couture ^(22)^, 1050 infants with DP more severe than type II received an average of 6.3 months of cranial-band therapy, and 82% of the infants reached type I or zero (no deformity). Branch ^(15)^ reported the results of helmet therapy on 4482 infants with DP, and the average Argenta classification improved by 3.2 after 5.7 months of treatment. Both studies reported that infants with a more severe type in Argenta classification resulted in lower correction and needed longer treatment time.

The effectiveness of helmet therapy in improving CVAI is also reported. Kluba ^(23)^ investigated 62 infants with severe DP. In their prospective longitudinal study, CVAI reduced from 13.3% to 4.1% after an average of 16 weeks of helmet therapy. Freudlsperger et al. ^(24)^ showed in a total of 213 infants that the mean initial CVAI of 9.8% reduced to 5.4% after an average of 4.5 months of helmet therapy. Here, our experience coincided with these previous results that helmet therapy improved CVAI of severe deformity.

On the other hand, Dutch randomized control trial ^(25)^ in 2014 discouraged the use of a helmet as a standard treatment for infants with moderate DP. It reported the equal effectiveness of helmet therapy and no treatment. However, this study was later criticized that the conclusion was misleading because they excluded patients with severe deformity, in whom the efficacy of treatment was most expected ^(12)^. Because of the poor design of the study, the conclusion of this randomized controlled trial must be viewed with caution.

### Influencing factors

Multivariate analysis revealed that, in general, patients with higher Argenta classification start with higher CA before helmet therapy, improve slowly, and end in higher CA at completion. CA after helmet therapy was suggested to be larger if the treatment is started at a later age and at a larger head circumference, although these two factors were not statistically significant. The treatment time was identified as the only statistically significant influencing factor of efficacy. This was true because most of the patients continued the therapy until their parents were satisfied with the head shape. Even with the same CA, if the Argenta was large before therapy, the correction tended to take time. Figure 3 must be interpreted with caution because this analysis was based only on two time points: measured at the start and completion of helmet therapy in each patient. In actual practice, most of the improvement takes place in the first 1-2 months, so the assumption that the slope has linearity would not be correct. Further analysis is planned to reflect this rate of improvement.

Individual analysis on the influencing factors was in accordance with the multivariate analysis. CA before and after helmet therapy tended to be larger in the group of patients who continued the use of the helmet for 21 weeks or longer. This was because patients with severe deformity accumulated in the longer-treating group.

CA after helmet therapy was larger in the group of patients who started helmet therapy at or later than 6 months of age. The deformity is thought to improve when the head grows. This implies that there is a less chance of improvement in head deformity in older patients because they have less potential for head growth. On the other hand, head circumference was not identified as influencing factor of efficacy. This was probably because head circumference was not a good indicator of the potential for head growth, whereas age was a stronger factor. As several lines of evidence have indicated, starting helmet therapy earlier will lead to faster and better correction of cranial asymmetry ^(13), (23), (24), (26), (27), (28)^.

CA after helmet therapy tended to be larger in the group with larger CA before therapy. This tendency must be kept in mind when introducing helmet therapy to patients.

### Safety

Almost all of the patients experienced increased sweating and skin rash, but these were well tolerated and managed by the parents. There were no other complications related to helmet therapy, including skin ulcer, and no complications related to the helmet that led to the cessation of the therapy, except for one patient with profound sweating.

The eight patients who were lost to follow-up may have experienced some complications, but detailed analysis on these patients revealed that most of the deformity had resolved at the timing of the last follow-up, and the possibility of a severe complication is unlikely.

Various authors reported that the complications were negligible ^(29), (30), (31), (32)^. Only van Wijk ^(25)^ reported a high incidence of adverse events like skin irritation (96%), augmented sweating (71%), unpleasant odor (76%), pain (33%), and feeling hindered from cuddling their child (77%) in their study. However, the high rate of fitting problems (73%) in the study made the quality of helmet therapy questionable ^(12)^. Because many of the complications reported by van Wijk ^(25)^ did not lead to the cessation of helmet therapy in our study, these should not be considered as adverse events. With a dedicated adjustment of the helmets, these complication rates should have been much lower, as shown in this study. Parents should be informed of increased sweating, skin rash, and the need for extra visit if any severe skin trouble is observed.

### Feasibility of the treatment in Japan

Twelve patients discontinued helmet therapy before completion. Eight patients were lost to follow-up, but most of the deformity had improved at the time of the last follow-up. One patient died because of congenital heart disease, and another had an operation for congenital atlantoaxial subluxation, both of which were not related to helmet therapy. In one patient, the mother chose to stop helmet therapy when she returned to work. However, the parents of many other patients had continued helmet therapy even after their return to work, and helmet therapy was well managed in nurseries. One patient with profound sweating discontinued the therapy, but this trouble was well managed by other parents.

Our study showed a low rate of adverse events along with a statistically significant improvement in DP. Helmet therapy is feasible in a clinical setting in Japan. Because orthotists are not allowed to work on patients unless there are specific instructions from medical doctors under Japanese law, the helmets need to be designed by the clinicians themselves or orthotists under close supervision of the clinicians. Our treatment system is now adapted by eight facilities with clinicians specializing in the treatment of skull deformities of infants.

Aihara et al. ^(13)^ previously reported the efficacy of helmet therapy in treating DP of Japanese infants. The study used an outcome index that was unique to their device and made it difficult to compare with other studies. The one-piece shell design of the helmet used in the study was difficult to adjust and needed multiple helmets before completion of the therapy. Our study adds another evidence to the safety, efficacy, and feasibility of the treatment with different types of helmet and outcome indices that are widely used. We believe that the two-piece shell design ^(20)^ of our helmet is superior in adapting to the growing head of the infants so that no extra helmet and cost are charged to the parents.

### Limitation and future direction

This was a single-arm, nonrandomized study without a control group of nontreated infants. To refuse helmet therapy for infants with severe deformity, who are typical candidates and have the possibility to achieve the greatest benefit, for randomization seems unethical.

Patients in the present study tended to be severe in plagiocephalic deformity and more brachycephalic compared to past reports. This was probably due to the cultural background of Japan where head deformity has not been focused on by pediatricians and has been generally accepted. Through the distribution of knowledge regarding the etiology and treatment of head deformity, earlier detection and an effective approach to head deformity are expected in the future.

This study focused on plagiocephaly and excluded pure brachycephaly without plagiocephalic deformity. However, a statistically significant improvement was observed in brachy-plagiocephaly, as shown in the normalization of the cephalic index. We are planning to report on the efficacy of helmet therapy on dolichocephaly and brachycephaly in the coming article.

The present study did not show the neuro-motor development of the infants before and after helmet therapy. We have collected the data in this study group, and we are planning to report the data in the near future.

## Article Information

### Conflicts of Interest

T Kaneko received honoraria for lectures from Medical U&A, Inc.

### Sources of Funding

This work was supported by Child Health and Development Research funding (institutional fund; grant number 21-14).

### Author Contributions

 Takamatsu and T Kaneko designed the study and wrote the initial draft of the manuscript. Hikosaka and Mikami contributed to the analysis and interpretation of data and assisted in the preparation of the manuscript. A Kaneko conceived the original idea. All authors approved the final version of the manuscript and agreed to be accountable for all aspects of the work in ensuring that questions related to the accuracy or integrity of any part of the work are appropriately investigated and resolved.

### Approval by Institutional Review Board(IRB)

No. 511 in the National Center for Child Health and Development
